# Supplementary material for: Comparison of standard and alternative methods for chest compressions in a single rescuer infant CPR: A prospective simulation study
Source: PLoS One. 2019 Dec 18;14(12):e0226632. doi: 10.1371/journal.pone.0226632 (PMC6919614; doi:10.1371/journal.pone.0226632)

In all figures, the post hoc analysis was performed by multiple-comparison testing using the Bonferroni method. A Bonferroni corrected p-value <0.05 was considered statistically significant.

Figure A. Comparison of rate of compression (/mm) between five methods.

Figure B. Comparison of correct rate (%) between five methods.

Figure C. Comparison of correct finger position (%) between five methods.

Figure D. Comparison of correct chest recoil (%) between five methods.

Figure E. Comparison of satisfaction score between five methods.

Figure F. Comparison of fatigue score between five methods.

Figure G. Comparison of easiness score between five methods.

TFT1: index-middle fingers; TFT2: middle-ring fingers; PT1: thumb-index-middle fingers; PT2: thumb-middle-ring fingers; PAT: thumb-index-middle fingers with plate.

a: statistically significant compared with TFT1, b: statistically significant compared with TFT2, c: statistically significant compared with PT1, d: statistically significant compared with PT2, e: statistically significant compared with PAT

**Figure A.**


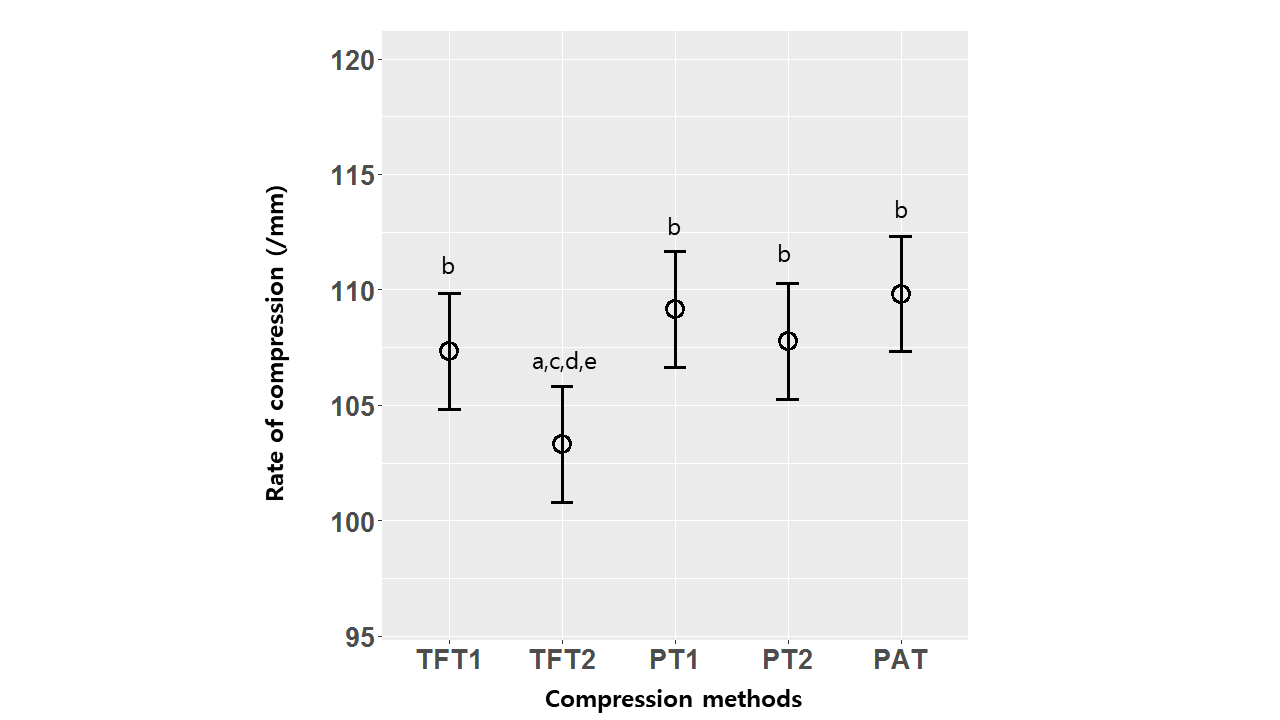


**Figure B.**


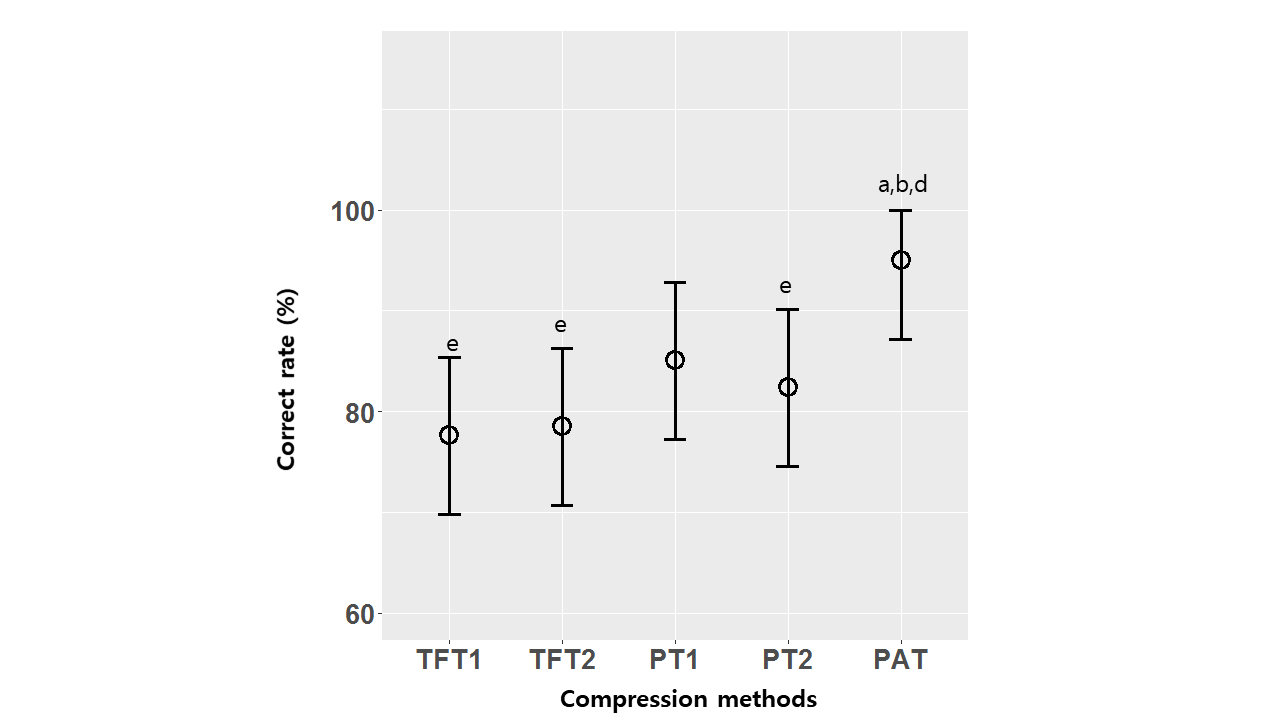


**Figure C.**
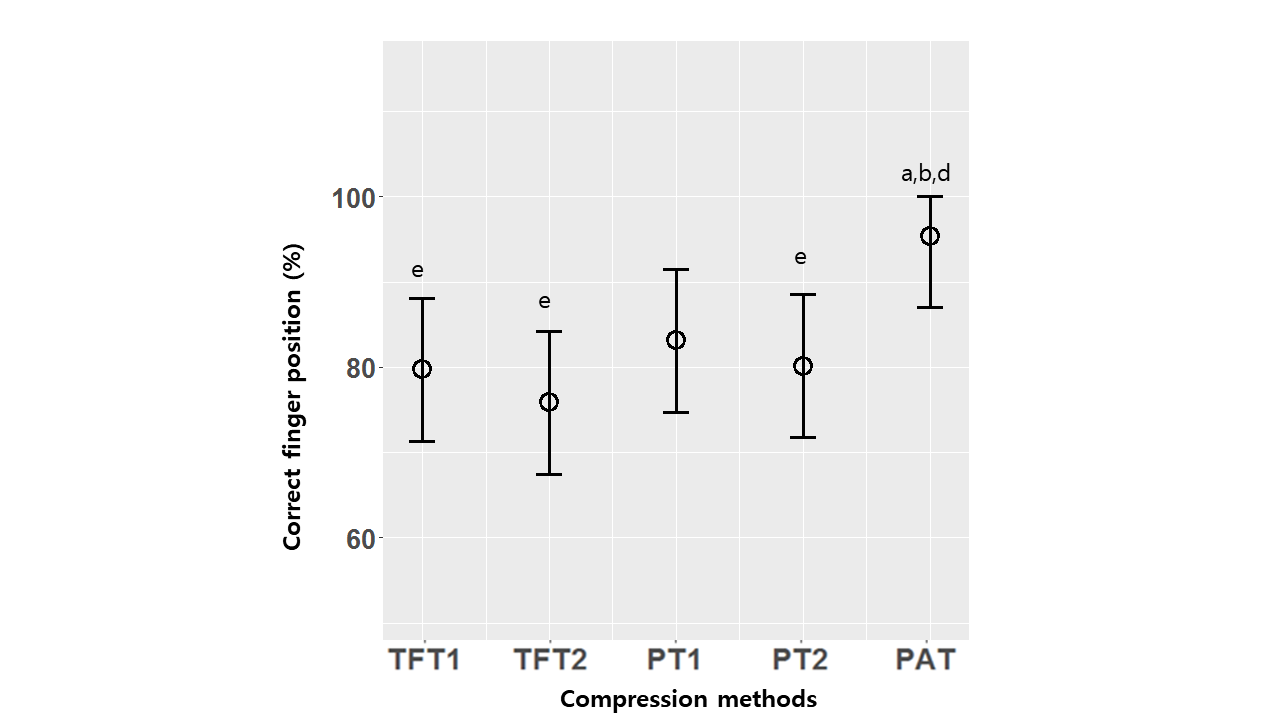


**Figure D.**
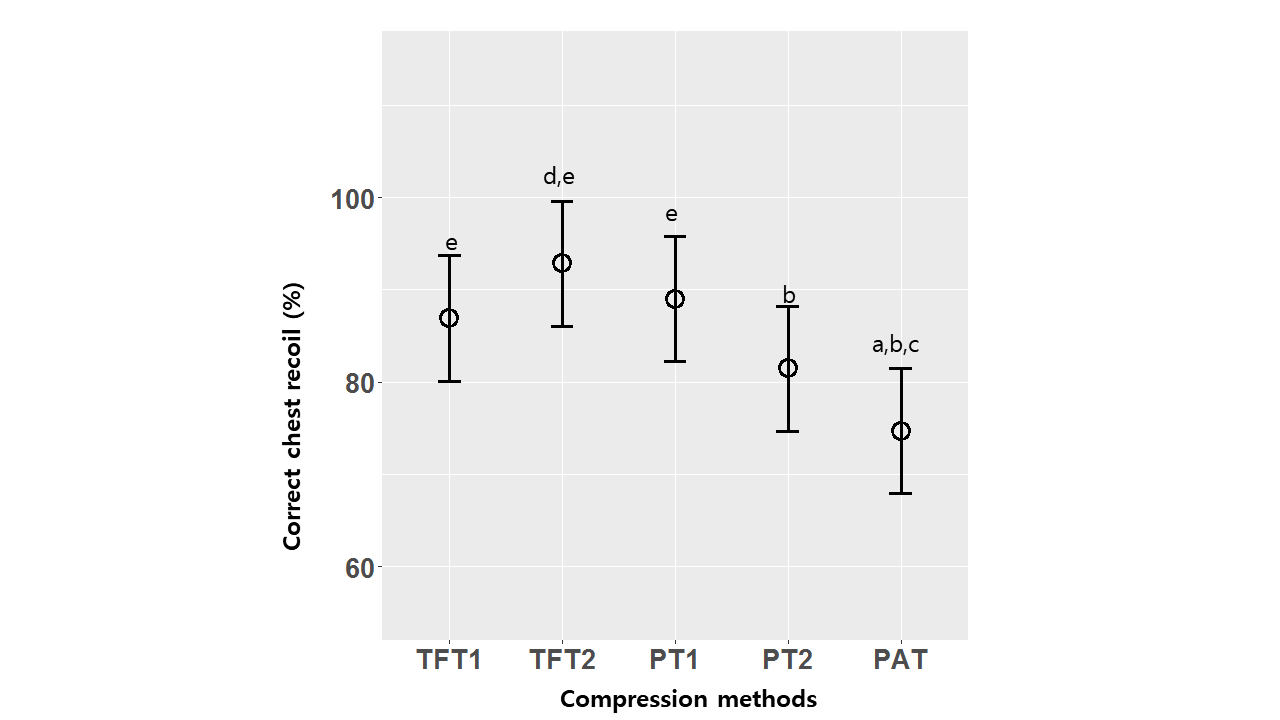


**Figure E.**
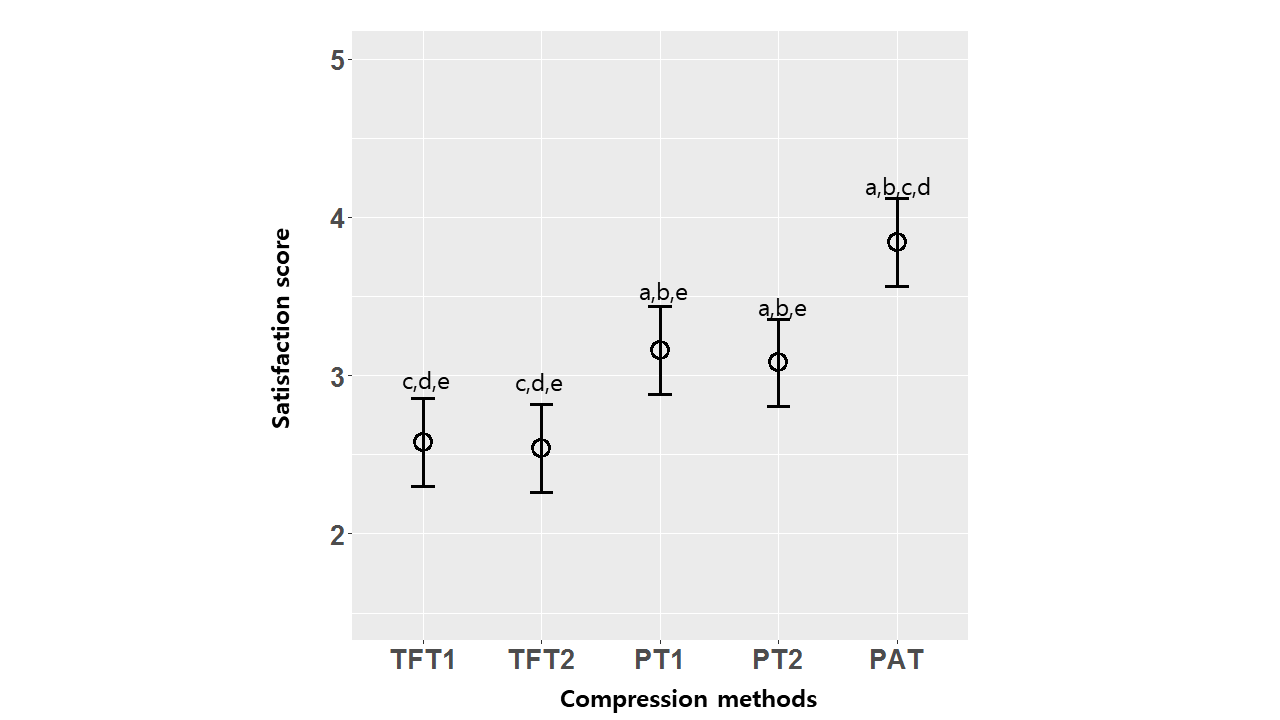


**Figure F.**
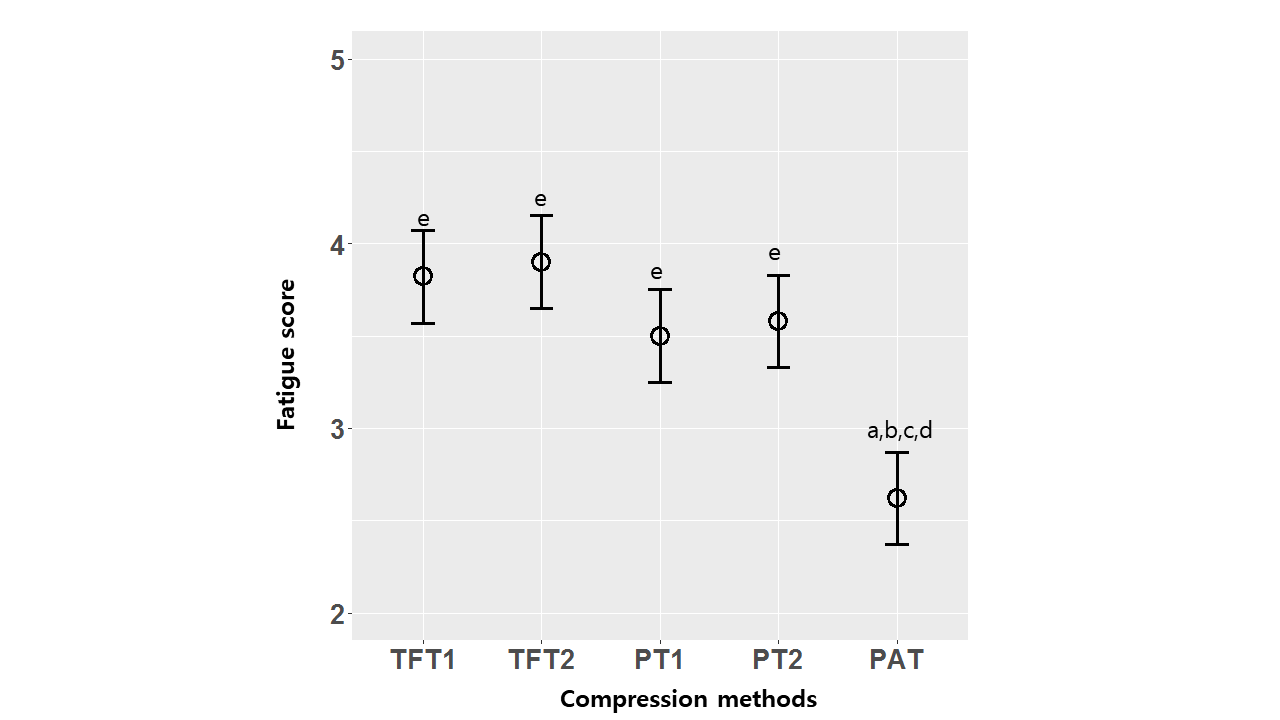


**Figure G.**
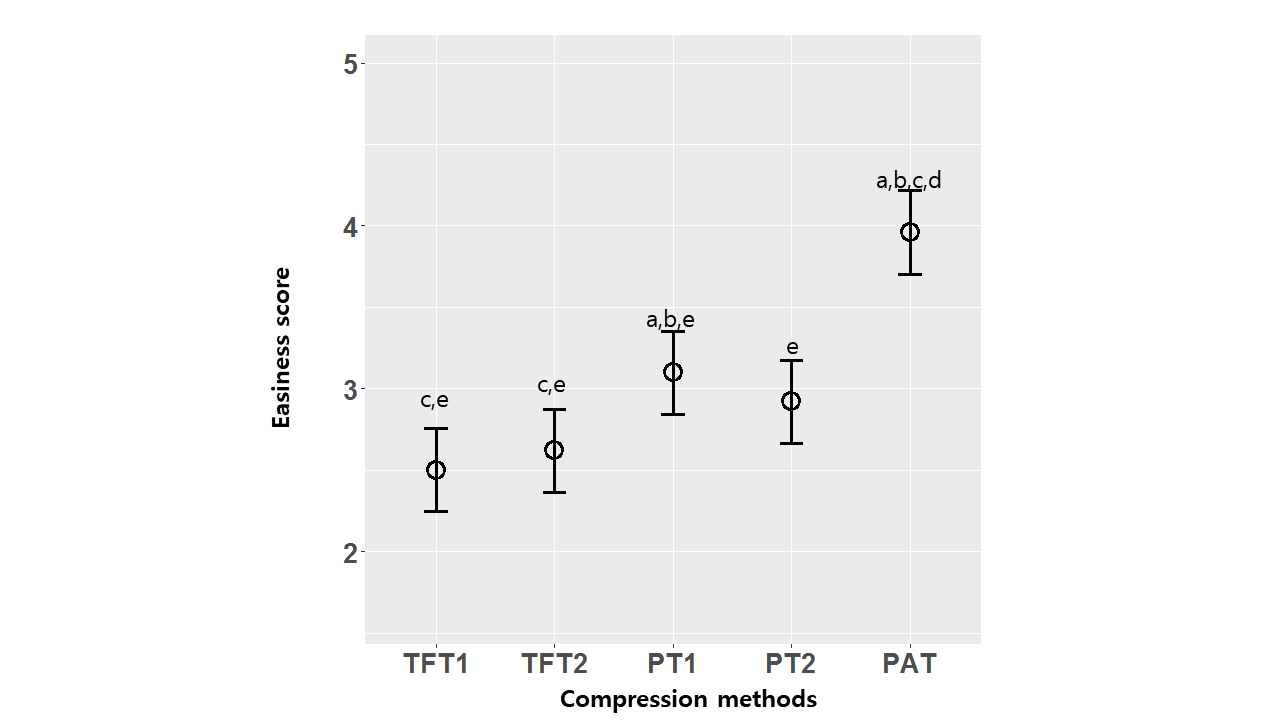

Supplement: S1 File — Figure A. Comparison of rate of compression (/mm) between five methods. Figure B. Comparison of correct rate (%) between five methods. Figure C. Comparison of correct finger position (%) between five methods. Figure D. Comparison of correct chest recoil (%) between five methods. Figure E. Comparison of satisfaction score between five methods. Figure F. Comparison of fatigue score between five methods. Figure G. Comparison of easiness score between five methods. (DOCX) [file pone.0226632.s001.docx]
