# Supplementary material for: Comparison of standard and alternative methods for chest compressions in a single rescuer infant CPR: A prospective simulation study
Source: PLoS One. 2019 Dec 18;14(12):e0226632. doi: 10.1371/journal.pone.0226632 (PMC6919614; doi:10.1371/journal.pone.0226632)
Supplement: Attachment — Submitted filename: Response to Reviewers - revised.docx [file pone.0226632.s002.docx]

**Response to Reviewers**

We would like to thank the reviewer for the careful and thorough reading of this manuscript and for the thoughtful comments and constructive suggestions.

**Comments to the Author**

1. Is the manuscript technically sound, and do the data support the conclusions?

The manuscript must describe a technically sound piece of scientific research with data that supports the conclusions. Experiments must have been conducted rigorously, with appropriate controls, replication, and sample sizes. The conclusions must be drawn appropriately based on the data presented.

Reviewer #1: Yes

Reviewer #2: Yes

________________________________________

2. Has the statistical analysis been performed appropriately and rigorously?

Reviewer #1: Yes

Reviewer #2: Yes

________________________________________

3. Have the authors made all data underlying the findings in their manuscript fully available?

The PLOS Data policy requires authors to make all data underlying the findings described in their manuscript fully available without restriction, with rare exception (please refer to the Data Availability Statement in the manuscript PDF file). The data should be provided as part of the manuscript or its supporting information, or deposited to a public repository. For example, in addition to summary statistics, the data points behind means, medians and variance measures should be available. If there are restrictions on publicly sharing data—e.g. participant privacy or use of data from a third party—those must be specified.

Reviewer #1: Yes

Reviewer #2: Yes

________________________________________

4. Is the manuscript presented in an intelligible fashion and written in standard English?

PLOS ONE does not copyedit accepted manuscripts, so the language in submitted articles must be clear, correct, and unambiguous. Any typographical or grammatical errors should be corrected at revision, so please note any specific errors here.

Reviewer #1: Yes

Reviewer #2: Yes

________________________________________

5. Review Comments to the Author

Please use the space provided to explain your answers to the questions above. You may also include additional comments for the author, including concerns about dual publication, research ethics, or publication ethics. (Please upload your review as an attachment if it exceeds 20,000 characters)

Reviewer #1: After reviewing the PONE-D-19-19476 manuscript entitled “Comparison of standard and alternative methods for chest compressions in a single rescuer infant CPR: a prospective simulation study” some suggestions and clarifications were raised as follows:

The aim of the study was to assess a new technique/device against 4 different techniques on a simulation manikin. In general, the manuscript is well written with minor spelling or grammar mistakes.

After checking the literature, and to my knowledge, this is the only manuscript that evaluated the use of a devise to assist the rescuers in performing CPR. This is a vital area of research since improving CPR in pediatric would rescue many lives.

Please find below some of the suggestions that would improve the quality of the manuscript.

Major suggestions or questions:

**1. Why did it take the investigators a long time after the simulation (Oct and Nov 2016) to prepare the manuscript and submit it for publications? Many papers were published during this period on new finger location and numbers since then. (1-5) one of the new techniques has many publications showing its superiority to TFT.**

**-** Thank you for your comment. I was able to finish the prospective study in a timely manner but due to personal health reasons, I was not able to finish writing the paper. Now that I have almost full recovered, I had the time to finally write it. I am aware that there were other papers published on new devices with a mannequin study, so I was deeply disappointed in my late publication. Thus, I would really love to have it published in your journal.

.

**2. The authors used a random cross over method to test the different techniques. Also, they mentioned that some of the techniques caused fatigue more than the others. Did the authors check the effect of starting with a harder technique on the performance of participants in the other techniques?**

**Response:** Thank you for your comment. We did not check the effect of starting with a harder technique because we purposely used a random order for the different chest compression techniques for each participant. Thus, we felt that starting with a harder technique was not necessary. Also, CPR was performed on the manikin for 2 minutes with each technique, followed by a 2-minute rest. This 2-minute rest time gave the participants ample time to recover from any fatigue.

**3. Did the authors check the difference in the performance of the participants depending on their levels (doctors, residents and students)?**

**Response:** Thank you for your comment. We did check the performance based upon their levels, but the data showed that it was not statistically significant. This point is already described in ‘Limitation’ session.

**4. How were the CD, the compression rate, finger position, and the recoil recorded and evaluated? Does the manikin record them?**

**-** Thank you for your comment. In the Study Design section of the Materials and Methods it is explained that a 3-month-old infant manikin (Resusci® Baby QCPR® by Laerdal Medical) was used and was fitted with a SimPad PLUS with Skill Reporter (Laerdal Medical). The chest compression performance data were collected using the SimPad Skill Reporter. For each technique, the total number of chest compressions (compression/min), CD (mm), and the percentages of correct CD, adequate compression rate, compression with correct finger position, and complete recoil of the chest were recorded. The analyzed comparison data among each different method groups are presented in Table 1.

**5. Does the manikin record other parameters like simulated SBP, DBP, mean arterial pressure, and pulse pressure?**

**-** Thank you for your comment. The manikin cannot record other parameters such as simulated SBP, DBP, mean arterial pressure, and pulse pressure. In the future, after the publication of this article, we plan to use an infant animal model to test the effectiveness of the new device and record other parameters.

**6. Did the author account for the primary/dominant hand of the participant? In the text they mentioned that all the participants used their right hand fingers for the compressions. How many participants were left handed? How was the performance of these participants in comparison with the right handed ones?**

**-** Thank you for your comment. Two of the participants were left-handed and were given the opportunity to use their dominant hand but both of them individually chose to use their right hand for chest compressions.

**7. The authors should provide further description or details about the used device in the PAT technique.**

**a. Is it patented?**

- The used device is not patented.

**b. Is it commercially available? What company invented it?**

- It is not commercially available. Three samples were made by the Department of Medical Engineering at Seoul National University as our request.

**c. Is there any other device in the market or experimental to compare this device to it?**

- There is no other device in the market or experimental to compare with this device.

**8. Depending on the author responses to question 7, the conflict of interest of the group would be more clear to the reviewers and the journal.**

- Thank you for your comment. We have added the above to the COI.

**Minor**

**1. Spelling mistakes (line 2 Paediatric and line 4, pediatric).**

- We made the spelling uniform.

**2. On line 145, remove one of “That” after TFT.**

- The word was removed.

**3. The description of the dimensions of the circular piece (line 39) must report either the diameter or the radius and not 2 dimensions since it is circle shape. Please modify.**

- We modified it to “a 1.0 cm in diameter…”

**4. Authors must improve the appearance of Table 1. It is hard to get the results as it is now.**

- The table has been modified.

**5. The orientation of the manikin and the fingers position in figure 1 (PT1 and PT2) is not clear. I suggest a better orientation like the other images.**

- Thank you for your comment. However, if we take pictures from another orientation, the position of thumb is not clearly visible so it is difficult to distinguish finger position.

**6. I suggest that the authors move some of the graphs presented in the supplementary sections to the main manuscript body to enrich the article.**

- Your suggestion has been noted. Among supplementary figures, two figures was moved as Fig 3.

**7. The numbering of the figures in the supporting information file should start with 1, and the authors should assign a special character to reflect the location of the figure in the supplement file. The authors should check the guidelines of PLoS one for this issue.**

- The numbering of the figures was adjusted according to the guidelines of PLoS one.

1. Ladny J, Smereka J, Rodriguez-Nunez A, Leung S, Ruetzler K, Szarpak L. Is there any alternative to standard chest compression techniques in infants? A randomized manikin trial of the new “2-thumb-fist” option. 2018. e9386 p.

2. Smereka J, Bielski K, Ladny J, Ruetzler K, Szarpak L. Evaluation of a newly developed infant chest compression technique A randomized crossover manikin trial 2017. e5915 p.

3. Smereka J, Szarpak L, Ladny J, Rodriguez-Nunez A, Ruetzler K. A Novel Method of Newborn Chest Compression: A Randomized Crossover Simulation Study 2018. 159 p.

4. Smereka J, Szarpak L, Rodriguez-Nunez A, Ladny J, Leung S, Ruetzler K. A randomized comparison of three chest compression techniques and associated hemodynamic effect during infant CPR: A randomized manikin study 2017. 1420-5 p.

5. Smereka J, Szarpak L, Smereka A, Leung S, Ruetzler K. 2017. Evaluation of new two-thumb chest compression technique for infant CPR performed by novice physicians. A randomized, crossover, manikin trial 604-9 p.

Reviewer #2: Dear authors,

Thank you for the opportunity to review the manuscript "Comparison of standard and alternative methods for chest compressions in a single rescuer infant CPR: a prospective simulation study".

A very interesting study concerning an important medical field that I have read with great interest.

I wish you all the best for publication.

Kind regards

________________________________________

6. PLOS authors have the option to publish the peer review history of their article (what does this mean?). If published, this will include your full peer review and any attached files.

If you choose “no”, your identity will remain anonymous but your review may still be made public.

Do you want your identity to be public for this peer review? For information about this choice, including consent withdrawal, please see our Privacy Policy.

Reviewer #1: Yes: Moustafa Al Hariri

Reviewer #2: No

[NOTE: If reviewer comments were submitted as an attachment file, they will be attached to this email and accessible via the submission site. Please log into your account, locate the manuscript record, and check for the action link "View Attachments". If this link does not appear, there are no attachment files to be viewed.]

While revising your submission, please upload your figure files to the Preflight Analysis and Conversion Engine (PACE) digital diagnostic tool, https://pacev2.apexcovantage.com/. PACE helps ensure that figures meet PLOS requirements. To use PACE, you must first register as a user. Registration is free. Then, login and navigate to the UPLOAD tab, where you will find detailed instructions on how to use the tool. If you encounter any issues or have any questions when using PACE, please email us at figures@plos.org. Please note that Supporting Information files do not need this step.
