# Supplementary material for: Comparison of standard and alternative methods for chest compressions in a single rescuer infant CPR: A prospective simulation study
Source: PLoS One. 2019 Dec 18;14(12):e0226632. doi: 10.1371/journal.pone.0226632 (PMC6919614; doi:10.1371/journal.pone.0226632)
Supplement: Attachment — Submitted filename: Response to Reviewers (minor revision).docx [file pone.0226632.s003.docx]

**Response to Reviewers**

Comments to the Author

1. If the authors have adequately addressed your comments raised in a previous round of review and you feel that this manuscript is now acceptable for publication, you may indicate that here to bypass the “Comments to the Author” section, enter your conflict of interest statement in the “Confidential to Editor” section, and submit your "Accept" recommendation.

Reviewer #2: (No Response)

Reviewer #3: (No Response)

2. Is the manuscript technically sound, and do the data support the conclusions?

The manuscript must describe a technically sound piece of scientific research with data that supports the conclusions. Experiments must have been conducted rigorously, with appropriate controls, replication, and sample sizes. The conclusions must be drawn appropriately based on the data presented.

Reviewer #2: (No Response)

Reviewer #3: Yes

3. Has the statistical analysis been performed appropriately and rigorously?

Reviewer #2: (No Response)

Reviewer #3: Yes

4. Have the authors made all data underlying the findings in their manuscript fully available?

The PLOS Data policy requires authors to make all data underlying the findings described in their manuscript fully available without restriction, with rare exception (please refer to the Data Availability Statement in the manuscript PDF file). The data should be provided as part of the manuscript or its supporting information, or deposited to a public repository. For example, in addition to summary statistics, the data points behind means, medians and variance measures should be available. If there are restrictions on publicly sharing data—e.g. participant privacy or use of data from a third party—those must be specified.

Reviewer #2: (No Response)

Reviewer #3: Yes

5. Is the manuscript presented in an intelligible fashion and written in standard English?

PLOS ONE does not copyedit accepted manuscripts, so the language in submitted articles must be clear, correct, and unambiguous. Any typographical or grammatical errors should be corrected at revision, so please note any specific errors here.

Reviewer #2: (No Response)

Reviewer #3: Yes

6. Review Comments to the Author

Please use the space provided to explain your answers to the questions above. You may also include additional comments for the author, including concerns about dual publication, research ethics, or publication ethics. (Please upload your review as an attachment if it exceeds 20,000 characters)

Reviewer #2: (No Response)

Reviewer #3: Dear authors,

I got to review your manuscript "Comparison of standard and alternative methods for chest compressions in a single rescuer infant CPR: a prospective simulation study", in which you show the superiority of a newly developed technique over the currently recommended ones. While in general, I think your manuscript is of great value and has gained a lot of value by the first revision, I would like to add a few minor points:

1.) Consider adding another figure about the currently recommended techniques (lines 8ff.) for the purpose of presentation.

**Thank you for your comment. The currently recommended one rescuer compression technique by the ERC and AHA guidelines is the 2-finger technique (TFT). TFT is shown as the first image in Figure 1 (TFT1).**

2.) I would appreciate a little more context about your motivation, on WHY you performed this research. You mention, that you wanted to "overcome the insufficient CD of the TFT and alleviate discomfort", however, please elaborate a little bit more, especially considering the fact THAT and why other techniques seem to be insufficient.

**Thank you for your comment. The manuscript was adjusted as following:**

**We undertook this study due to TFT having insufficient CD and to alleviate discomfort in the user’s fingers after prolonged compressions. TTHT was reported to consistently achieve higher blood and coronary perfusion pressure and greater compression depth (CD) in a variety of animal and manikin models (7-11). However, most resuscitation guidelines recommend the TFT method for single-rescuer infant CPR because of the longer hands-off time and difficulty of postural change**.

3.) In line 51, you should mention that the 5-point Likert scale questionnaire was performed after EVERY one of the techniques. As it is written now, it appears that it was performed just ones at the very end (In line 118 you mention it correctly).

**Thank you for your comment. Line 51 was changed to the following:**

**At the end of each technique, the participants completed the 5-point Likert scale-based questionnaire.**

4.) In the measured outcomes section you write about all the outcomes, however, it remains unclear how "correct" is defined. in Data Collection you write about the correct finger position, but how correct (or adequate) compression rate, correct chest recoil and correct CD are defined, remains unclear. Please clarify.

**Thank you for your comment.**

**Lines 65 – 68 were adjusted to include the word “correct” as follows:**

**The correct finger position was specified as just below the nipple line in the middle of the chest in a 1.0 × 1.0 cm square. We defined correct CD as ≥ 40 mm, correct compression rate as 100–120/min, and complete recoil as the manikin’s chest returning to its original position.**

5.) I would be very interested, whether the sequence yielded different results for the different techniques (e.g. has the TFT1 technique yielded better results when performed first...?) I am wondering, because I can imagine, that when performing five different techniques, the motivation reduces over time. Furthermore, it would be also very interesting how the "correctness" of each technique changed over the duration of the performance - can any statement concerning the recommended length before a change of rescucitator be given? How significant is the fatigueness during the performance?

**Thank you for your comment. We did not check the effect of starting with a harder technique because we purposely used a random order for the different chest compression techniques for each participant. Thus, we felt that starting with a harder technique was not necessary. Also, chest compressions were performed on the manikin for 2 minutes with each technique, followed by a 2-minute rest. This 2-minute rest time gave the participants ample time to recover from any fatigue. A method of chest compression for 2-minute is widely recommended before changing the resuscitator.**

6.) Please add a sentence to the limitations section that this experiment should also be repeated with a group of experienced doctors/health care practitioners (like paediatricians or emergency doctors) to evaluate the effectiveness in a group of other relevance.

**Thank you for your comment. This sentence was added to the Limitations section:**

**Finally,** **for future studies to evaluate the effectiveness of the techniques, the participants should include experienced doctors and healthcare practitioners since the current study only included medical students and residents.**

Thank you.

7. PLOS authors have the option to publish the peer review history of their article (what does this mean?). If published, this will include your full peer review and any attached files.

If you choose “no”, your identity will remain anonymous but your review may still be made public.

Do you want your identity to be public for this peer review? For information about this choice, including consent withdrawal, please see our Privacy Policy.

Reviewer #2: No

Reviewer #3: No

[NOTE: If reviewer comments were submitted as an attachment file, they will be attached to this email and accessible via the submission site. Please log into your account, locate the manuscript record, and check for the action link "View Attachments". If this link does not appear, there are no attachment files to be viewed.]

While revising your submission, please upload your figure files to the Preflight Analysis and Conversion Engine (PACE) digital diagnostic tool, https://pacev2.apexcovantage.com/. PACE helps ensure that figures meet PLOS requirements. To use PACE, you must first register as a user. Registration is free. Then, login and navigate to the UPLOAD tab, where you will find detailed instructions on how to use the tool. If you encounter any issues or have any questions when using PACE, please email us at figures@plos.org. Please note that Supporting Information files do not need this step.

- A rebuttal letter that responds to each point raised by the academic editor and reviewer(s). This letter should be uploaded as separate file and labeled 'Response to Reviewers'.
- A marked-up copy of your manuscript that highlights changes made to the original version. This file should be uploaded as separate file and labeled 'Revised Manuscript with Track Changes'.
- An unmarked version of your revised paper without tracked changes. This file should be uploaded as separate file and labeled 'Manuscript'.

Please note while forming your response, if your article is accepted, you may have the opportunity to make the peer review history publicly available. The record will include editor decision letters (with reviews) and your responses to reviewer comments. If eligible, we will contact you to opt in or out.

We look forward to receiving your revised manuscript.

Kind regards,

Lars-Peter Kamolz, M.D., Ph.D., M.Sc.
Academic Editor
PLOS ONE


[Note: HTML markup is below. Please do not edit.]

Reviewers' comments:

Reviewer's Responses to Questions

**Comments to the Author**

1. If the authors have adequately addressed your comments raised in a previous round of review and you feel that this manuscript is now acceptable for publication, you may indicate that here to bypass the “Comments to the Author” section, enter your conflict of interest statement in the “Confidential to Editor” section, and submit your "Accept" recommendation.

Reviewer #2: (No Response)

Reviewer #3: (No Response)

2. Is the manuscript technically sound, and do the data support the conclusions?

The manuscript must describe a technically sound piece of scientific research with data that supports the conclusions. Experiments must have been conducted rigorously, with appropriate controls, replication, and sample sizes. The conclusions must be drawn appropriately based on the data presented.

Reviewer #2: (No Response)

Reviewer #3: Yes

3. Has the statistical analysis been performed appropriately and rigorously?

Reviewer #2: (No Response)

Reviewer #3: Yes

4. Have the authors made all data underlying the findings in their manuscript fully available?

The [PLOS Data policy](http://www.plosone.org/static/policies.action#sharing) requires authors to make all data underlying the findings described in their manuscript fully available without restriction, with rare exception (please refer to the Data Availability Statement in the manuscript PDF file). The data should be provided as part of the manuscript or its supporting information, or deposited to a public repository. For example, in addition to summary statistics, the data points behind means, medians and variance measures should be available. If there are restrictions on publicly sharing data—e.g. participant privacy or use of data from a third party—those must be specified.

Reviewer #2: (No Response)

Reviewer #3: Yes

5. Is the manuscript presented in an intelligible fashion and written in standard English?

PLOS ONE does not copyedit accepted manuscripts, so the language in submitted articles must be clear, correct, and unambiguous. Any typographical or grammatical errors should be corrected at revision, so please note any specific errors here.

Reviewer #2: (No Response)

Reviewer #3: Yes

6. Review Comments to the Author

Please use the space provided to explain your answers to the questions above. You may also include additional comments for the author, including concerns about dual publication, research ethics, or publication ethics. (Please upload your review as an attachment if it exceeds 20,000 characters)

Reviewer #2: (No Response)

Reviewer #3: Dear authors,
I got to review your manuscript "Comparison of standard and alternative methods for chest compressions in a single rescuer infant CPR: a prospective simulation study", in which you show the superiority of a newly developed technique over the currently recommended ones. While in general, I think your manuscript is of great value and has gained a lot of value by the first revision, I would like to add a few minor points:
1.) Consider adding another figure about the currently recommended techniques (lines 8ff.) for the purpose of presentation.
2.) I would appreciate a little more context about your motivation, on WHY you performed this research. You mention, that you wanted to "overcome the insufficient CD of the TFT and alleviate discomfort", however, please elaborate a little bit more, especially considering the fact THAT and why other techniques seem to be insufficient.
3.)In line 51, you should mention that the 5-point Likert scale questionnaire was performed after EVERY one of the techniques. As it is written now, it appears that it was performed just ones at the very end (In line 118 you mention it correctly).
4.) In the measured outcomes section you write about all the outcomes, however, it remains unclear how "correct" is defined. in Data Collection you write about the correct finger position, but how correct (or adequate) compression rate, correct chest recoil and correct CD are defined, remains unclear. Please clarify.
5.) I would be very interested, whether the sequence yielded different results for the different techniques (e.g. has the TFT1 technique yielded better results when performed first...?) I am wondering, because I can imagine, that when performing five different techniques, the motivation reduces over time. Furthermore, it would be also very interesting how the "correctness" of each technique changed over the duration of the performance - can any statement concerning the recommended length before a change of rescucitator be given? How significant is the fatigueness during the performance?
6.) Please add a sentence to the limitations section that this experiment should also be repeated with a group of experienced doctors/health care practitioners (like paediatricians or emergency doctors) to evaluate the effectiveness in a group of other relevance.

Thank you.

7. PLOS authors have the option to publish the peer review history of their article ([what does this mean?](https://journals.plos.org/plosone/s/editorial-and-peer-review-process#loc-peer-review-history)). If published, this will include your full peer review and any attached files.


If you choose “no”, your identity will remain anonymous but your review may still be made public.


**Do you want your identity to be public for this peer review?** For information about this choice, including consent withdrawal, please see our [Privacy Policy](https://www.plos.org/privacy-policy).

Reviewer #2: No

Reviewer #3: No


[NOTE: If reviewer comments were submitted as an attachment file, they will be attached to this email and accessible via the submission site. Please log into your account, locate the manuscript record, and check for the action link "View Attachments". If this link does not appear, there are no attachment files to be viewed.]


While revising your submission, please upload your figure files to the Preflight Analysis and Conversion Engine (PACE) digital diagnostic tool, <https://pacev2.apexcovantage.com/>. PACE helps ensure that figures meet PLOS requirements. To use PACE, you must first register as a user. Registration is free. Then, login and navigate to the UPLOAD tab, where you will find detailed instructions on how to use the tool. If you encounter any issues or have any questions when using PACE, please email us at [figures@plos.org](mailto:figures@plos.org). Please note that Supporting Information files do not need this step.

PONE-D-19-19476R1
Comparison of standard and alternative methods for chest compressions in a single rescuer infant CPR: a prospective simulation study
PLOS ONE

Dear Dr Kim,

Thank you for submitting your manuscript to PLOS ONE. After careful consideration, we feel that it has merit but does not fully meet PLOS ONE’s publication criteria as it currently stands. Therefore, we invite you to submit a revised version of the manuscript that addresses the points raised during the review process.

We would appreciate receiving your revised manuscript by Dec 15 2019 11:59PM. When you are ready to submit your revision, log on to https://www.editorialmanager.com/pone/ and select the 'Submissions Needing Revision' folder to locate your manuscript file.

If you would like to make changes to your financial disclosure, please include your updated statement in your cover letter.

To enhance the reproducibility of your results, we recommend that if applicable you deposit your laboratory protocols in protocols.io, where a protocol can be assigned its own identifier (DOI) such that it can be cited independently in the future. For instructions see: <http://journals.plos.org/plosone/s/submission-guidelines#loc-laboratory-protocols>

Please include the following items when submitting your revised manuscript:

- A rebuttal letter that responds to each point raised by the academic editor and reviewer(s). This letter should be uploaded as separate file and labeled 'Response to Reviewers'.
- A marked-up copy of your manuscript that highlights changes made to the original version. This file should be uploaded as separate file and labeled 'Revised Manuscript with Track Changes'.
- An unmarked version of your revised paper without tracked changes. This file should be uploaded as separate file and labeled 'Manuscript'.

Please note while forming your response, if your article is accepted, you may have the opportunity to make the peer review history publicly available. The record will include editor decision letters (with reviews) and your responses to reviewer comments. If eligible, we will contact you to opt in or out.

We look forward to receiving your revised manuscript.

Kind regards,

Lars-Peter Kamolz, M.D., Ph.D., M.Sc.
Academic Editor
PLOS ONE


[Note: HTML markup is below. Please do not edit.]

Reviewers' comments:

Reviewer's Responses to Questions

**Comments to the Author**

1. If the authors have adequately addressed your comments raised in a previous round of review and you feel that this manuscript is now acceptable for publication, you may indicate that here to bypass the “Comments to the Author” section, enter your conflict of interest statement in the “Confidential to Editor” section, and submit your "Accept" recommendation.

Reviewer #2: (No Response)

Reviewer #3: (No Response)

2. Is the manuscript technically sound, and do the data support the conclusions?

The manuscript must describe a technically sound piece of scientific research with data that supports the conclusions. Experiments must have been conducted rigorously, with appropriate controls, replication, and sample sizes. The conclusions must be drawn appropriately based on the data presented.

Reviewer #2: (No Response)

Reviewer #3: Yes

3. Has the statistical analysis been performed appropriately and rigorously?

Reviewer #2: (No Response)

Reviewer #3: Yes

4. Have the authors made all data underlying the findings in their manuscript fully available?

The [PLOS Data policy](http://www.plosone.org/static/policies.action#sharing) requires authors to make all data underlying the findings described in their manuscript fully available without restriction, with rare exception (please refer to the Data Availability Statement in the manuscript PDF file). The data should be provided as part of the manuscript or its supporting information, or deposited to a public repository. For example, in addition to summary statistics, the data points behind means, medians and variance measures should be available. If there are restrictions on publicly sharing data—e.g. participant privacy or use of data from a third party—those must be specified.

Reviewer #2: (No Response)

Reviewer #3: Yes

5. Is the manuscript presented in an intelligible fashion and written in standard English?

PLOS ONE does not copyedit accepted manuscripts, so the language in submitted articles must be clear, correct, and unambiguous. Any typographical or grammatical errors should be corrected at revision, so please note any specific errors here.

Reviewer #2: (No Response)

Reviewer #3: Yes

6. Review Comments to the Author

Please use the space provided to explain your answers to the questions above. You may also include additional comments for the author, including concerns about dual publication, research ethics, or publication ethics. (Please upload your review as an attachment if it exceeds 20,000 characters)

Reviewer #2: (No Response)

Reviewer #3: Dear authors,
I got to review your manuscript "Comparison of standard and alternative methods for chest compressions in a single rescuer infant CPR: a prospective simulation study", in which you show the superiority of a newly developed technique over the currently recommended ones. While in general, I think your manuscript is of great value and has gained a lot of value by the first revision, I would like to add a few minor points:
1.) Consider adding another figure about the currently recommended techniques (lines 8ff.) for the purpose of presentation.
2.) I would appreciate a little more context about your motivation, on WHY you performed this research. You mention, that you wanted to "overcome the insufficient CD of the TFT and alleviate discomfort", however, please elaborate a little bit more, especially considering the fact THAT and why other techniques seem to be insufficient.
3.)In line 51, you should mention that the 5-point Likert scale questionnaire was performed after EVERY one of the techniques. As it is written now, it appears that it was performed just ones at the very end (In line 118 you mention it correctly).
4.) In the measured outcomes section you write about all the outcomes, however, it remains unclear how "correct" is defined. in Data Collection you write about the correct finger position, but how correct (or adequate) compression rate, correct chest recoil and correct CD are defined, remains unclear. Please clarify.
5.) I would be very interested, whether the sequence yielded different results for the different techniques (e.g. has the TFT1 technique yielded better results when performed first...?) I am wondering, because I can imagine, that when performing five different techniques, the motivation reduces over time. Furthermore, it would be also very interesting how the "correctness" of each technique changed over the duration of the performance - can any statement concerning the recommended length before a change of rescucitator be given? How significant is the fatigueness during the performance?
6.) Please add a sentence to the limitations section that this experiment should also be repeated with a group of experienced doctors/health care practitioners (like paediatricians or emergency doctors) to evaluate the effectiveness in a group of other relevance.

Thank you.

7. PLOS authors have the option to publish the peer review history of their article ([what does this mean?](https://journals.plos.org/plosone/s/editorial-and-peer-review-process#loc-peer-review-history)). If published, this will include your full peer review and any attached files.


If you choose “no”, your identity will remain anonymous but your review may still be made public.


**Do you want your identity to be public for this peer review?** For information about this choice, including consent withdrawal, please see our [Privacy Policy](https://www.plos.org/privacy-policy).

Reviewer #2: No

Reviewer #3: No


[NOTE: If reviewer comments were submitted as an attachment file, they will be attached to this email and accessible via the submission site. Please log into your account, locate the manuscript record, and check for the action link "View Attachments". If this link does not appear, there are no attachment files to be viewed.]


While revising your submission, please upload your figure files to the Preflight Analysis and Conversion Engine (PACE) digital diagnostic tool, <https://pacev2.apexcovantage.com/>. PACE helps ensure that figures meet PLOS requirements. To use PACE, you must first register as a user. Registration is free. Then, login and navigate to the UPLOAD tab, where you will find detailed instructions on how to use the tool. If you encounter any issues or have any questions when using PACE, please email us at [figures@plos.org](mailto:figures@plos.org). Please note that Supporting Information files do not need this step.

PONE-D-19-19476R1
Comparison of standard and alternative methods for chest compressions in a single rescuer infant CPR: a prospective simulation study
PLOS ONE

Dear Dr Kim,

Thank you for submitting your manuscript to PLOS ONE. After careful consideration, we feel that it has merit but does not fully meet PLOS ONE’s publication criteria as it currently stands. Therefore, we invite you to submit a revised version of the manuscript that addresses the points raised during the review process.

We would appreciate receiving your revised manuscript by Dec 15 2019 11:59PM. When you are ready to submit your revision, log on to https://www.editorialmanager.com/pone/ and select the 'Submissions Needing Revision' folder to locate your manuscript file.

If you would like to make changes to your financial disclosure, please include your updated statement in your cover letter.

To enhance the reproducibility of your results, we recommend that if applicable you deposit your laboratory protocols in protocols.io, where a protocol can be assigned its own identifier (DOI) such that it can be cited independently in the future. For instructions see: <http://journals.plos.org/plosone/s/submission-guidelines#loc-laboratory-protocols>

Please include the following items when submitting your revised manuscript:

- A rebuttal letter that responds to each point raised by the academic editor and reviewer(s). This letter should be uploaded as separate file and labeled 'Response to Reviewers'.
- A marked-up copy of your manuscript that highlights changes made to the original version. This file should be uploaded as separate file and labeled 'Revised Manuscript with Track Changes'.
- An unmarked version of your revised paper without tracked changes. This file should be uploaded as separate file and labeled 'Manuscript'.

Please note while forming your response, if your article is accepted, you may have the opportunity to make the peer review history publicly available. The record will include editor decision letters (with reviews) and your responses to reviewer comments. If eligible, we will contact you to opt in or out.

We look forward to receiving your revised manuscript.

Kind regards,

Lars-Peter Kamolz, M.D., Ph.D., M.Sc.
Academic Editor
PLOS ONE


[Note: HTML markup is below. Please do not edit.]

Reviewers' comments:

Reviewer's Responses to Questions

**Comments to the Author**

1. If the authors have adequately addressed your comments raised in a previous round of review and you feel that this manuscript is now acceptable for publication, you may indicate that here to bypass the “Comments to the Author” section, enter your conflict of interest statement in the “Confidential to Editor” section, and submit your "Accept" recommendation.

Reviewer #2: (No Response)

Reviewer #3: (No Response)

2. Is the manuscript technically sound, and do the data support the conclusions?

The manuscript must describe a technically sound piece of scientific research with data that supports the conclusions. Experiments must have been conducted rigorously, with appropriate controls, replication, and sample sizes. The conclusions must be drawn appropriately based on the data presented.

Reviewer #2: (No Response)

Reviewer #3: Yes

3. Has the statistical analysis been performed appropriately and rigorously?

Reviewer #2: (No Response)

Reviewer #3: Yes

4. Have the authors made all data underlying the findings in their manuscript fully available?

The [PLOS Data policy](http://www.plosone.org/static/policies.action#sharing) requires authors to make all data underlying the findings described in their manuscript fully available without restriction, with rare exception (please refer to the Data Availability Statement in the manuscript PDF file). The data should be provided as part of the manuscript or its supporting information, or deposited to a public repository. For example, in addition to summary statistics, the data points behind means, medians and variance measures should be available. If there are restrictions on publicly sharing data—e.g. participant privacy or use of data from a third party—those must be specified.

Reviewer #2: (No Response)

Reviewer #3: Yes

5. Is the manuscript presented in an intelligible fashion and written in standard English?

PLOS ONE does not copyedit accepted manuscripts, so the language in submitted articles must be clear, correct, and unambiguous. Any typographical or grammatical errors should be corrected at revision, so please note any specific errors here.

Reviewer #2: (No Response)

Reviewer #3: Yes

6. Review Comments to the Author

Please use the space provided to explain your answers to the questions above. You may also include additional comments for the author, including concerns about dual publication, research ethics, or publication ethics. (Please upload your review as an attachment if it exceeds 20,000 characters)

Reviewer #2: (No Response)

Reviewer #3: Dear authors,
I got to review your manuscript "Comparison of standard and alternative methods for chest compressions in a single rescuer infant CPR: a prospective simulation study", in which you show the superiority of a newly developed technique over the currently recommended ones. While in general, I think your manuscript is of great value and has gained a lot of value by the first revision, I would like to add a few minor points:
1.) Consider adding another figure about the currently recommended techniques (lines 8ff.) for the purpose of presentation.
2.) I would appreciate a little more context about your motivation, on WHY you performed this research. You mention, that you wanted to "overcome the insufficient CD of the TFT and alleviate discomfort", however, please elaborate a little bit more, especially considering the fact THAT and why other techniques seem to be insufficient.
3.)In line 51, you should mention that the 5-point Likert scale questionnaire was performed after EVERY one of the techniques. As it is written now, it appears that it was performed just ones at the very end (In line 118 you mention it correctly).
4.) In the measured outcomes section you write about all the outcomes, however, it remains unclear how "correct" is defined. in Data Collection you write about the correct finger position, but how correct (or adequate) compression rate, correct chest recoil and correct CD are defined, remains unclear. Please clarify.
5.) I would be very interested, whether the sequence yielded different results for the different techniques (e.g. has the TFT1 technique yielded better results when performed first...?) I am wondering, because I can imagine, that when performing five different techniques, the motivation reduces over time. Furthermore, it would be also very interesting how the "correctness" of each technique changed over the duration of the performance - can any statement concerning the recommended length before a change of rescucitator be given? How significant is the fatigueness during the performance?
6.) Please add a sentence to the limitations section that this experiment should also be repeated with a group of experienced doctors/health care practitioners (like paediatricians or emergency doctors) to evaluate the effectiveness in a group of other relevance.

Thank you.

7. PLOS authors have the option to publish the peer review history of their article ([what does this mean?](https://journals.plos.org/plosone/s/editorial-and-peer-review-process#loc-peer-review-history)). If published, this will include your full peer review and any attached files.


If you choose “no”, your identity will remain anonymous but your review may still be made public.


**Do you want your identity to be public for this peer review?** For information about this choice, including consent withdrawal, please see our [Privacy Policy](https://www.plos.org/privacy-policy).

Reviewer #2: No

Reviewer #3: No


[NOTE: If reviewer comments were submitted as an attachment file, they will be attached to this email and accessible via the submission site. Please log into your account, locate the manuscript record, and check for the action link "View Attachments". If this link does not appear, there are no attachment files to be viewed.]


While revising your submission, please upload your figure files to the Preflight Analysis and Conversion Engine (PACE) digital diagnostic tool, <https://pacev2.apexcovantage.com/>. PACE helps ensure that figures meet PLOS requirements. To use PACE, you must first register as a user. Registration is free. Then, login and navigate to the UPLOAD tab, where you will find detailed instructions on how to use the tool. If you encounter any issues or have any questions when using PACE, please email us at [figures@plos.org](mailto:figures@plos.org). Please note that Supporting Information files do not need this step.
